# Pediatric Chemotherapy Drugs Associated With Cardiotoxicity

**DOI:** 10.7759/cureus.19658

**Published:** 2021-11-17

**Authors:** Gazala Hitawala, Esha Jain, Lisett Castellanos, Radhika Garimella, Radhika Akku, Adila K Chamavaliyathil, Huma Irfan, Vikash Jaiswal, Jonathan Quinonez, Maher Dakroub, Muhammad Hanif, Ali H Baloch, Ivan S Gomez, John Dylewski

**Affiliations:** 1 Internal Medicine, Jersey City (JC) Medical Center, Orlando, USA; 2 Medicine, American University of Antigua, St. John's, ATG; 3 Pediatrics, Larkin Community Hospital, South Miami, USA; 4 Research, Larkin Community Hospital, South Miami, USA; 5 Internal Medicine, California Institute of Behavioral Neurosciences & Psychology, Fairfield, USA; 6 Pediatrics, Ras Al Khaimah (RAK) Medical and Health Sciences University, Ras Al Khaimah, ARE; 7 Medicine, Larkin Community Hospital, Miami, USA; 8 Neurology/Osteopathic Neuromuscular Medicine, Larkin Community Hospital, Miami, USA; 9 Hematology and Oncology, Larkin Community Hospital, South Miami, USA; 10 Internal Medicine, Khyber Medical College Peshawar, Hayatabad Medical Complex, Peshawar, PAK; 11 Research, University of Maryland Medical Center, Baltimore, USA; 12 Cardiology, Larkin Community Hospital, South Miami, USA

**Keywords:** dexrazoxane, cardiotoxic agents, pediatric cancer, chemotherapy associated cardiotoxicity, anthracycline

## Abstract

Pediatric cancers are a common cause of childhood morbidity. As a result, chemotherapeutic regimens have been designed to target childhood cancers. These medications are necessary to treat pediatric cancers, however, oncology management options are accompanied by multiple negative and potentially fatal adverse effects. Although anthracyclines are the most commonly used chemotherapeutic agents associated with cardiotoxicity, we also explore other chemotherapeutic drugs used in children that can potentially affect the heart. Genetic variations resulting in single nucleotide polymorphism (SNP) have the propensity to modify the cardiotoxic effects of the chemotherapy drugs. The clinical presentation of the cardiac effects can vary from arrhythmias and heart failure to completely asymptomatic. A range of imaging studies and laboratory investigations can protect the heart from severe outcomes. The physiology of the heart and the effect of drugs in children vary vividly from adults; therefore, it is crucial to study the cardiotoxic effect of chemotherapy drugs in the pediatric population. This review highlights the potential contributing factors for cardiotoxicity in the pediatric population and discusses the identification and management options.

## Introduction and background

Cancer is rapidly progressive and fatal. It does not spare genders, races, or ages. Oncology treatment has developed immensely over the years and will continue to do so. With any pharmaceutical treatment, there are always adverse effects clinicians must be aware of so that they can aim to prevent or decrease the progression of these effects. The authors of this paper intend to analyze various chemotherapy regimens used to treat common pediatric malignancies. We focus on the class of anthracyclines, tyrosine kinase inhibitors, and VEGF inhibitors. Further, we will investigate possible therapies that can help lessen the adverse effect of cardiotoxicity from chemotherapeutic drugs in this population.

A leading cause of morbidity and mortality in the pediatric population (ages 0-19) is cancer. Approximately 400,000 pediatric patients under 19 years old are diagnosed with cancer on a yearly basis [1]. The accurate estimate of the incidence of pediatric cancer globally is difficult to establish as certain countries lack cancer registries. Even if the registries do exist, the true incidence is usually underestimated as children with cancer often go undiagnosed [2].

Some of the more common pediatric cancers include leukemias, cancers of the soft tissues, and nervous system, with acute lymphoblastic leukemia (ALL) being the most commonly diagnosed pathology in the pediatric population [3]. Within the United States of America (USA), more than 15,000 people under the age of 19 are diagnosed with cancer annually. Advancements in medical care and the introduction of novel chemotherapy treatments have allowed for an increase in the rate of cancer survivors from around 50% to 80% [4]. Children have successfully been living longer. However, as the number of long-term survivors increases, so has premature heart disease. Heart disease has now become a driving cause of death in the pediatric population due to the cardiotoxic side effects of chemotherapy. The North American Childhood Cancer Survivor Study (CCSS) indicated no significant improvement in the cumulative incidence of associated cardiac pathologies such as heart failure and ischemic heart disease since the 1970s [5]. Cardiotoxicity can be defined by analyzing the change in the left ventricular shortening fraction (LVSF) diagnosed via echocardiogram (ECHO), or as a clinical diagnosis showing apparent heart failure in the patient. If LVSF is lower than 28%, or if there is more than 10% change in LVSF from baseline, this constitutes cardiotoxicity [6].

To top the list of potent chemotherapeutic regimens in treating childhood cancers are the class of anthracyclines. These chemotherapy drugs have treated more than 50% of childhood cancers. Other notable chemotherapeutic yet cardiotoxic regimens include tyrosine kinase inhibitors like imatinib and dasatinib [7]. Functionally, cardiomyocytes encompass a decreased ability in cellular regeneration and are more susceptible to adverse effects of anthracyclines, notably doxorubicin [8]. Adriamycin (ADR) is one of the more potent chemotherapeutic agents in treating pediatric malignancies, including ALL, and has led to improved rates of survival. However, it has been proven to cause cardiac arrhythmias and ultimately congestive heart failure (CHF). Studies have shown the important role that carvedilol (nonselective beta-blocker) can play as a cardio-protectant drug. It displays potent antioxidant effects and anti-apoptotic properties on the heart [9]. Therapies such as Dexrazoxane have been used since the 1980s for their role as a cardioprotective therapy towards anthracycline-related cardiotoxicity [10]. Introduced in the 2000s, this drug has been shown to be efficacious in decreasing the incidence of CHF and left ventricular heart failure [6]. Exploring cardioprotective regimens to combat the adverse effects of cardiotoxicity from chemotherapy is crucial for improved outcomes in children living with cancer.

Unfortunately, while highly therapeutic, anthracycline and other chemotherapy drug exposure can cause significant cardiotoxicity in children. The severity of toxic adverse effects on the heart ranges from mild cardiac dysfunction to cardiomyopathy and CHF [11]. We must ask ourselves, what are some, if any, ways we can prevent the progression of cardiotoxic effects? Is there potential to mitigate the damage caused by other preventative therapies? Targeting anthracycline-related cardiotoxicity before it develops into chronic complications like CHF can be crucial in mitigating the level of cardiotoxicity in children as they age. Studies have shown that cancer survivors in the pediatric population exhibit a long latency between asymptomatic cardiomyopathy and symptomatic CHF [9].

This article investigates the more commonly used chemotherapeutic drugs in the pediatric population, the pathophysiology behind its cardiotoxic effects, and most importantly, what can we do about it? Analyzing various treatments is important; combination therapy can prove to be successful, whether it is adding cardioprotective therapies or combining physical rehabilitation. The discovery of new approaches to treating the pediatric population can hopefully lead to longer survival outcomes.

## Review

Pathophysiology of cardiotoxicity associated with peds chemotherapy

The pediatric cellular composition of the heart is intricately different from that of the adult heart. Thus, understanding the cellular differences in the pediatric heart allows clinicians to understand the mechanism of impact and subsequent recovery of chemotherapy on the cardiomyocyte. The fetal and neonatal heart highly express positive cell cycle regulators like cyclin-dependent kinases and cyclins, which are not enunciated in the heart of an adult [12]. Furthermore, another mechanism involved in cell proliferation is telomerase activity. In neonatal cardiomyocytes, the telomerase activity facilitates S-phase entry and suppression of cyclin-dependent kinase inhibitors which induces cell proliferation [13,14], unlike in the adult heart. Understanding that the pediatric heart functions differently versus the adult heart, we must use different strategies to combat chemotherapy-related cardiotoxicity.

The most widely studied chemotherapeutic drugs which are strongly associated with cardiotoxicity are anthracyclines. During DNA replication, the DNA topoisomerase relieves overwinding of DNA by introducing DNA breaks. The chemotherapeutic drug anthracyclines target topoisomerase II (TOP2) which introduces double-stranded DNA breaks [15]. Furthermore, there are 2 isoenzymes of TOP2 in humans. Anthracyclines target both the isoenzymes of TOP2 (TOP2A and TOP2B) and stabilize the TOP2 DNA complex. TOP2A mediates the antineoplastic effect of the anthracyclines, and TOP2B is responsible for the cardiotoxicity associated with anthracyclines. To support this further, studies on mice and human embryonic stem cells have proven that disruption of the TOP2B prevented the cardiotoxicity associated with anthracyclines [16,17]. A spectrum of genetic variations, specifically SNP is identified which result in changes in the mitochondrial functioning, signal transduction, cellular transport, and cell cycle regulation that promote the cardiotoxic effects of the anthracyclines, known as anthracycline-associated cardiotoxicity (ACT).

The DNA regulatory sequence, retinoic acid receptor gamma variant (RARG) binds to the TOP2B promoter to regulate the gene expression. A study on 280 pediatric oncology patients identified single nucleotide polymorphism (SNP) rs2229774 in RARG. This change resulted in the decreased repression of the TOP2B, which consequently resulted in greater cardiomyocyte death [18]. Doxorubicinol, a metabolite of doxorubicin (anthracycline), directly inhibits the Ryanodine receptor (RYR2) and the Ca+2-ATPase (SERCA2A). As a result, it disrupts both the replenishment of the calcium stores and calcium release by the sarcoplasmic reticulum [19]. Moreover, doxorubicinol binds the F1F0 proton pump in the mitochondria of the cardiomyocyte, which leads to reduced energy production [20]. In a cohort study on 170 cases and 317 controls, the polymorphism in carbonyl reductase (rs1056892) led to a tripled risk of cardiomyopathy with anthracyclines [21]. The carbonyl reductase metabolite (CBR3) with SNP (rs1056892) is strongly associated with ACT [22].

Studies on mice have revealed that the ATP binding cassette subfamily C member 1 (ABCC1) is elicited in higher proportions following exposure to doxorubicin [23]. These proteins are transporters that are bound to the membrane and require energy from ATP hydrolysis for clearance of substrates from the cells [24]. Another study performed on the pediatric population diagnosed with ALL revealed that the nucleotide polymorphism rs3743527 that occurs within the 3’ UTR of ABCC1 decreased the posttranscriptional gene expression which resulted in ACT [25]. A retrospective cohort study also identified an association of SNP in ABCC2 with four times the increased risk of ACT [26]. Moreover, a decrease in LV ejection fraction and LVSF were associated with SNP rs7627754, which had a TT genotype in the promoter region of ABCC5 [27].

The variant (rs10426377) of sulfotransferase family cytosolic member 2B1 (SULT2B1), which increases the solubility of drugs in water and promotes renal excretion was also found to be associated with ACT. On stratification, it also revealed that the variant affected only men [28,29]. It is hypothesized that the variant decreases the metabolization and excretion of anthracycline by the kidneys. However, more research must be performed to establish this cause [11].

The oxidative metabolism in the cardiomyocytes causes the production of reactive oxygen species (ROS). It is evident that exposure to ROS leads to the terminal differentiation of the cardiomyocytes [30]. Anthracyclines produce superoxide anions (ROS) through two mechanisms - pathway of the electron transport chain and pathway with redox cycling of iron‐anthracycline complexes [11]. The anthracycline is a lipophilic molecule that can easily diffuse through the membrane. Once it reaches inside the inner mitochondrial membrane, it binds to the cardiolipin and converts to semiquinone form by nicotinamide adenine dinucleotide. The product, anthracycline semiquinone subsequently reverts to the quinone form by releasing ROS and bypassing the electron transport chain [31]. These ROS are the contributors to lipid peroxidation, resulting in membrane damage and the release of intracellular proteins like LDH and cardiac troponins [32]. Moreover, the endomyocardial biopsies showcased myocyte vacuolization and myofibrillar lysis in ACT [33,34].

In the early stages of a failing heart, the heart attempts to compensate via NADPH. NADPH generates ROS in order to induce hypertrophy of the heart. The SNP rs13058338 in the RAC2 subunit of NADPH oxidase has been correlated with ACT susceptibility. Additionally, NADPH oxidase knockout mice were safeguarded from heart failure caused by doxorubicin, and NADPH oxidase inhibitors demonstrated a reduction in the damage of cardiomyocytes when exposed to anthracyclines [35,36]. This solidifies that NADPH can play a pertinent role in the ACT.

In a study on childhood ALL, the participants were reported to have inactivated SNPs in SOD2, CAT, GSTT1, and GSTM1 [37]. When undergoing oxidative stress, the superoxide dismutase II (SOD2) converts superoxide to hydrogen peroxide, which is then converted into water by catalase (CAT), thus preventing the conversion to hydroxyl free radical. Glutathione S transferase (GSTT1 and GSTM1) prevents damaging interactions with molecules like DNA, protein, and lipids by conjugating free glutathione to chemotherapeutic free radical drug metabolites. The SNP rs10836235 in CAT was found to be associated with ACT resistance. It was speculated that this SNP resulted in interference in the binding of a negative regulator of CAT transcription. This caused increased expression of CAT, which protected from ROS and eventually from ACT [37]. On the other hand, the variant of glutathione S transferase (GSTP1) with the SNP rs1695 was concomitant with cardiotoxicity in a study on children with osteosarcoma. Reduced activity of GSTP1 further left the cardiomyocyte unprotected from ROS, leading to susceptibility to ACT [38].

The missense mutation rs12468485 in G protein-coupled receptor 35 (GPCR35) has an association with increased risk as well as increased severity of ACT [39]. A study showed that GPR35 exposition is perceptive to hypoxia in cardiomyocytes and its overexpression results in a shortened viability period [40]. Hyaluronan is a glycosaminoglycan that serves as scaffolds during tissue remodeling. This glycosaminoglycan is synthesized with the help of hyaluronan synthase‐3 (HAS3). The SNP rs2232228 in HAS3 was found to manipulate the risk of ACT [41]. A case-controlled cohort study recruited children that have survived cancer, with and without cardiomyopathy after anthracycline exposure. The patients with the AA genotype in SNP rs2232228 were at an approximately ninefold higher risk of developing ACT versus those with the GG genotype [41]. Hyaluronan is also found to increase the survival of rat cardiomyocytes from ROS damage, which further establishes that discrepancy with HAS3 leading to alteration in hyaluronan may increase the risk of cardiotoxicity [42].

Some acquired and congenital pathologies like hypertension (HTN) and hereditary hemochromatosis are also found to be associated with ACT. Hypertension is found to defy danger for developing ACT in long-term childhood cancer survivors. In a study on 108 childhood cancer survivors, 12 hypertension susceptibility loci were identified. However, two variants PLCE1 rs932764 and ATP2B1 rs17249754 were reported to be protective against ACT development [43]. Phospholipase C ε (PLCE1) is involved in intracellular signaling. PLCE1 activates protein kinase D that reduces the generation of ROS. Thus, this variant of PLCE1 possibly protects against the ROS generated by the anthracyclines. Miranda et al. hypothesized that hereditary hemochromatosis possibly plays a role in making patients susceptible to ACT. They established that HFE deficient mice who were treated with doxorubicin were found to contain increased levels of creatine kinase in the serum, thus reflecting damage to the cardiac tissue [44]. Furthermore, to explore this aspect in childhood cancer patients, Lipshultz et al. determined two SNP in HFE correlated with ACT in 184 pediatric patients. Out of these, 10% carried SNP rs1800562 that demonstrated an increase in cardiac troponin T and reduced LV function at 2.2-year follow‐up [45]. 

**Figure 1:**
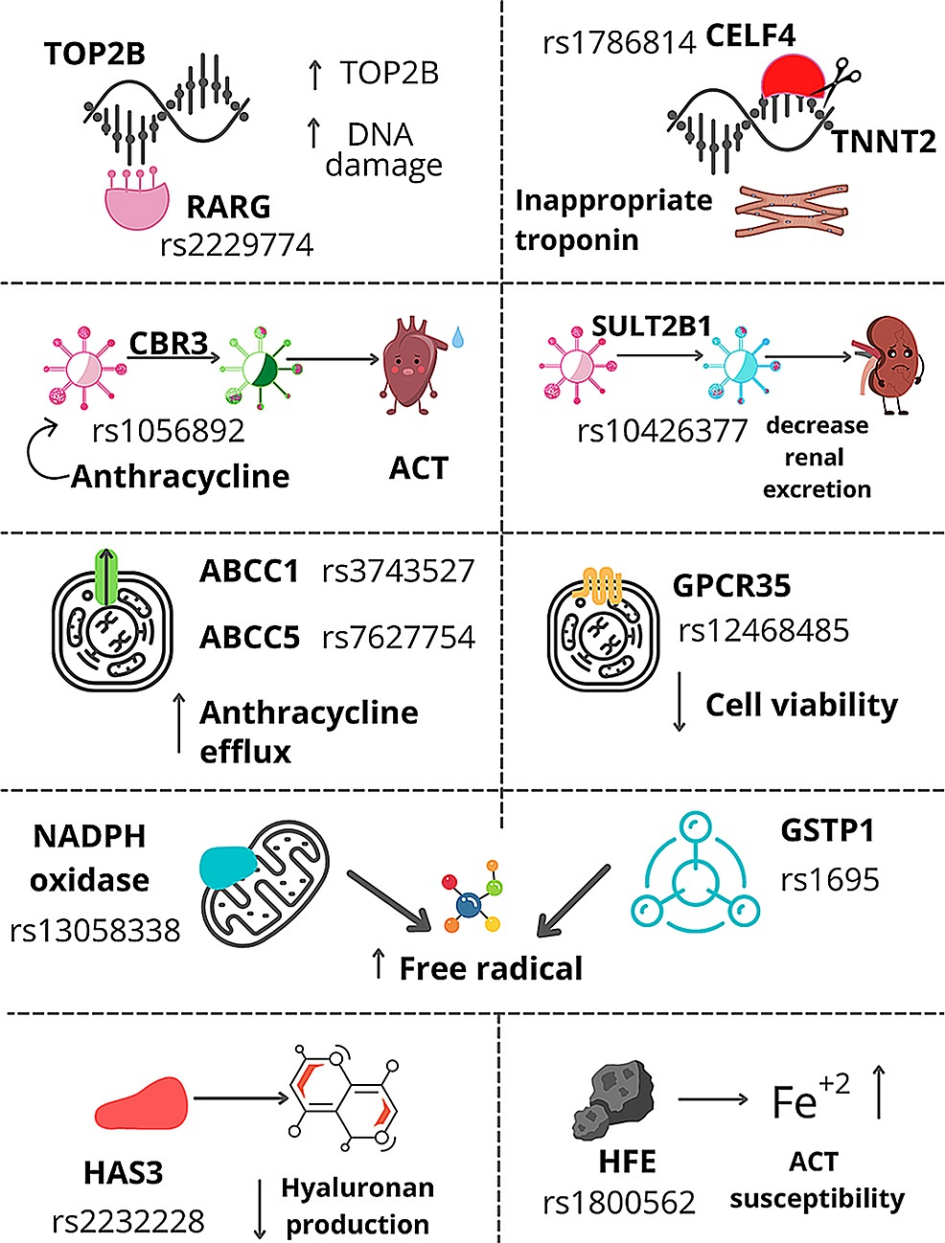
Single nucleotide polymorphism (SNP) increasing ACT risk ACT - anthracycline-associated cardiotoxicity

The CUGBP Elav‐like family member 4 (CELF4) is an RNA binding protein that regulates the alternative splicing of the gene TNNT2. This gene codes for cardiac troponin T, which is found within the thin filaments of the sarcomeres [46]. The embryonic heart predominantly expresses the variants of cardiac troponin T with an alternate exon 5, which is down-regulated significantly in the hearts of the adult population. The CELF4 variant has decreased affinity to the TNNT2 in the adult heart, and compromises the contractility and eventually LV ejection fraction [47]. Wang et al. established an association of SNP rs1786814 in CELF4 in childhood cancer survivors to the propensity to develop cardiomyopathy post-anthracyclines. Exposure to more than 300 mg/m^2^ of an anthracycline with the CC genotype in CELF4 rs1786814 had a 10 times higher risk of progression to ACT, while the genotype with CT and TT attenuated the risk of ACT [46]. Figure 1 shows the various SNP that results in an increased risk of ACT [11].

Apart from anthracyclines, it is worth exploring the cardiac complication mechanism of other chemotherapeutic drugs which are under trial or are actively being used for the management of cancers in the pediatric population. Paclitaxel is a vinca alkaloid that was found to be effective for the management of recurrent childhood brain tumors [48]. The taxanes act by interrupting microtubule formation. In the adult population, a broad spectrum of cardiac manifestations, including bradycardia, cardiac ischemia, atrioventricular block, ventricular arrhythmias has been found to be associated with taxanes. Cremophor EL (CrEL) is a formulation vehicle used for paclitaxel. The CrEL is known to interfere with various biological effects, one among them is hypersensitivity reaction [49]. It is suspected that the release of histamine due to CrEL could be the causative in the cardiovascular manifestation of paclitaxel. The cardiac H1 and H2 receptors are found to be associated with increased oxygen demand, coronary vasoconstriction, and chronotropic effects [50]. As the microtubules do not play a significant role in myocardial functioning, it is less likely that the microtubular impact of taxanes is responsible for cardiac abnormalities. However, the microtubular disruption can definitely impact the subcellular organelle which can indirectly impact the myocardium [51]. As paclitaxel is becoming a prospective management option for pediatric tumors, the cardiotoxic effect in the adult population should be an alert for cautious use in children [48].

Tyrosine kinase inhibitors are well established to treat a variety of pediatric cancers like CML, AML, ALL, GIST, neuroblastoma, renal cell carcinoma, etc. [52]. Tyrosine kinase inhibitors act by various mechanisms like interfering with the ATP binding site of the enzyme or blocking the ligand-binding site, to hinder the activity of the enzyme tyrosine kinase [52]. The trigger for the cardiotoxic effect of tyrosine kinase inhibitors is the activation of the endoplasmic reticulum (ER) stress response. Although this response is in place as a protective mechanism, the prolonged activation can further lead to the activation of the pro-death pathways. A study on humans and mice revealed that dose-dependant imatinib use caused destitution of the membrane potential which causes the release of the cytochrome c into the cytosol. It also reported decreased ATP, increased activity of caspase 3 and 7, and the dominant finding of cytosolic vacuolization which marked the occurrence of necrotic death [53]. Thus, the overactivation of the ER stress response facilitates both apoptotic and necrotic death of the cardiac tissue. 

The procreation of diverse solid tumors is reliant on the VEGF, which leads to the proliferation of blood vessels. However, the VEGF also produces cytokines that inhibit apoptosis and promote malignancy. In vitro studies have reported bevacizumab represses the VEGF-mediated angiogenesis in Wilms tumor, neuroblastoma, hepatoblastoma, and rhabdomyosarcoma [54-57]. It is hypothesized that the VEGF-mediated angiogenesis and endothelial maintenance protect the cardiomyocyte from oxidative damage, which is disrupted by bevacizumab. Alternatively, the cardiotoxicity is correlated to its propensity to induce HTN by inhibiting NO synthase resulting in vasoconstriction, which eventually causes LV dysfunction [58]. As there are ongoing clinical trials for the use of bevacizumab in refractory solid tumors, it is worth paying attention to its potential cardiotoxic side effects. 

Since 1994, 5 fluorouracil (5 FU) has been under trial for the management of a spectrum of childhood solid tumors [59]. Although the pathophysiology of the association of 5FU with its cardiotoxic effect is unclear, there are various hypotheses to support the cause. The most commonly suggested underlying mechanisms are coronary artery thrombosis, arteritis, and vasospasm [60,61]. However, in some patients angiography failed to elicit vasospasm. Moreover, in the same study, vasodilators prior to 5 FU infusions were not successful in preventing angina [62]. The other proposed hypotheses are direct toxicity on the myocardium, autoimmune response, or by-products generated in the alkaline solution of 5 FU vials during storage [60]. As the drug has long been under trial to explore its potential in the management of pediatric tumors, it is worth paying attention to the cardiac manifestation of 5 FU in children.

Alkylating agents like cyclophosphamide are also used as chemotherapeutic agents. More importantly, it is commonly used during bone marrow transplantation in children. In the 1986 study by Goldberg et al., it was concluded that cyclophosphamide resulted in dose-related cardiotoxicity in children [63]. The metabolites of cyclophosphamide are thought to result in increased oxidative stress and direct injury to the endothelium. This causes extravasation of the fluids and proteins resulting in edema, hemorrhage, and microthrombi. Cardiac tissue autopsy in cyclophosphamide toxicity revealed extravasation of blood in the myocardium, deposition of fibrin in the interstitium, and capillary microthrombi. Furthermore, the presence of intra-mitochondrial inclusion, hyper contraction bands, myofibrillar damage, and fibrin deposition in cytoplasm supported the presence of myocardial necrosis [64]. The pathophysiology pertaining to the discussed chemotherapeutic drugs is necessary to understand as clinicians can be more prudent with children at higher risk, and can develop management options that avoid detrimental outcomes in children. 

Clinical presentation of cardiotoxicity associated with peds chemotherapy 

There is an enhanced risk of cardiotoxicity with the commonly used anthracyclines (doxorubicin or daunorubicin). Based on the time of presentation, the symptoms are classified into acute and chronic. The acute cardiotoxic symptoms associated with anthracyclines usually occur within hours of infusion during the treatment course. These symptoms primarily include arrhythmias; at a higher dose, heart failure, myocarditis, and pericarditis occur. Although acute symptoms usually resolve with stopping treatment, cardiac function declines over time [4]. On the other hand, chronic symptoms generally present months to years post-therapy. These symptoms likely occur due to decreased contractility of myocardiocytes and an increased left ventricle afterload [65]. The most common manifestation primarily occurs due to decreased thickness of the left ventricular wall, resulting in decreased cardiac function, and eventually progressing to CHF [66]. Although CHF can occur with any dose of anthracycline, studies reveal that there is an enhanced risk with cumulative doses of anthracycline, specifically doses more than 300mg/m^2^ [66]. In a study conducted by Trachtenberg et al., pediatric patients managed with anthracycline for ALL originally presented with dilated cardiomyopathy, decreased left ventricle contractility, and overall cardiac dilation. Over time, this pattern changed to restrictive cardiomyopathy with decreased left ventricle contractility and wall thickness [67]. 

The cardiotoxic adverse events associated with cyclophosphamide usually present 14 days post-therapy and include myocarditis and CHF. These side effects typically occur with high doses of cyclophosphamide and rarely occur with low doses [68]. Similarly, CHF, as well as arrhythmias, can occur with ifosfamide. The presentation of these symptoms usually occurs 6-23 days after therapy and is associated with high doses. Cytarabine administration primarily results in the development of atrial and ventricular arrhythmias as well as CHF [69]. Cisplatin can cause a spectrum of cardiotoxic adverse events, including myocardial infarction, and arrhythmias such as atrial fibrillation, supraventricular tachycardia, and left bundle branch block. Therapy with cisplatin results in decreased levels of calcium and magnesium, therefore, leading to arrhythmias; combining cisplatin with other chemotherapy agents further increases this risk of the development of arrhythmias. Supplemental magnesium and calcium can be beneficial to restore normal levels of these electrolytes [65]. The most common cardiac-related adverse event linked with paclitaxel treatment is asymptomatic bradycardia. In a study conducted by Rowinsky et al. during a 24-hour continuous period of cardiac monitoring, bradycardia occurred in 29% of patients [51]. Also, tyrosine kinase inhibitors such as imatinib and sunitinib are associated with cardiotoxic symptoms such as left ventricular dysfunction, heart failure, and arrhythmias [69].

Pediatric patients on treatment with these chemotherapeutic agents must be monitored closely during therapy as well as post-therapy. The primary goal of monitoring is to identify cardiotoxic changes early in the course so that the patient’s treatment plan can be modified accordingly and further decrease the risk of developing severe cardiac disease. 

Monitoring

Cardiac Biomarkers Importance and EKG

Monitoring the deleterious effects of chemotherapy involves a multifactorial approach. Cardiac-specific biomarkers are used by clinicians in detecting early cardiovascular injuries. These biomarkers include cardiac troponins, specifically measuring serum concentrations of cardiac troponin T in the pediatric population. A study performed on 134 children found elevated concentrations of cardiac troponin T in over a third of the children who were managed with anthracyclines for high-risk ALL. Another commonly measured cardiac biomarker to detect ventricular wall stress is the N-terminal pro-brain natriuretic peptide (NT-proBNP). This biomarker has been used to predict cardiotoxicity from anthracycline exposure in children suffering from ALL. Lipshultz et al. described that NT-proBNP concentrations were notably increased in greater than 90% of a group of 156 children, especially in the first 90 days of therapy, when given anthracycline pharmacotherapy at a moderate dose. This finding was associated with long-term abnormal LV wall thickness suggesting pathologic remodeling of the LV [70].

Electrocardiograms (ECGs) are an efficient way to screen for the risk of arrhythmias and potential conduction pathologies, such as prolonged QT intervals [71]. Identifying abnormal cardiac rhythms earlier in their course can help to minimize damage to the cardiac myocytes. ECGs are beneficial in monitoring cardiotoxic adverse events associated with these chemotherapy drugs. They are useful as they are non-invasive and inexpensive. In addition to revealing the electrical activity of the heart, ECGs also indicate signs of cardiomyopathies. However, ECGs are limited as they can not be utilized to determine left ventricular ejection fraction [72]. Additionally, as a monitoring tool, they only offer clinicians an idea of cardiac function at one point in time. The use of 24-Holter monitors can perhaps provide a better and more accurate idea of the changes in cardiac conduction. This method can prove to aid in the recognition of events leading to palpitations or syncope for a longer period of time [71].

Echocardiogram

Multiple modalities exist in regards to imaging and monitoring the function of the cardiac system. Clinicians have developed an accurate, reproducible, and minimally invasive imaging modality called the ECHO. ECHO has now become the mainstay imaging modality for serial monitoring of the heart for potential cardiotoxic effects from chemotherapy [73]. The 2D-ECHO is the most widely used imaging technique for evaluating cardiotoxicity in children, before, during, and after chemotherapeutic treatment. Not only is it minimally invasive, but also the ECHO curtails exposure to radiation and is safe to use in patients with comorbidities such as kidney disease. ECHO allows clinicians to assess the LV volume, dimensions, and overall function. Furthermore, the ECHO allows for visualization of any valvular or pericardial pathologies [74]. Due to the increased prevalence of cardiac complications with chemotherapy in the pediatric population, monitoring throughout the course of chemotherapy treatment is essential and can be useful in mitigating long-term cardiovascular toxicity. 

ECHOs provide an accurate estimation of the ejection fraction along with the systolic and diastolic function of the heart [72]. The study by Altena et al. proved that the most sensitive indicator for detecting early changes in cardiac function was the change in diastolic function [72]. Thus, ECHO is definitely a helpful strategy for accurately combating early cardiotoxic effects associated with chemotherapeutic drugs [72].

A more sensitive study to detect the damage to the myocardial tissue is myocardial strain imaging. It measures the global longitudinal strain (GLS), which helps in the early detection of LV dysfunction [75]. A study reported that while only 5.8% of the childhood cancer survivors had reduced ejection fraction, the GLS was found to be decreased in 32% of the survivors, which helped clinicians to be more vigilant towards these patients [76]. Another study reported a reduction in the longitudinal strain (LS) over time which was independent of body size [75]. Thus, continuous monitoring overtime for these patients should be considered. Moreover, a decreasing pattern of LS over time could be more helpful in identifying cardiotoxicity in childhood cancer survivors rather than just relying on a single LS value [77].

Cardiac MRI

Cardiac MRI provides the unique ability to characterize myocardial tissue without an invasive biopsy. Additionally, it allows the recognition of early cardiac injury without the harmful effects of ionizing radiation. Cardiac MRI is used for the measurement of left ventricular mass which helps in the evaluation of late cardiotoxicity [78]. In a 2013 study, out of 62 childhood cancer survivors, 79% were reported with left ventricular dysfunction and 80% with right ventricular dysfunction [79]. In another study, monitoring with cardiac MRI in childhood cancer survivors revealed that 48% of the survivors had LV mass >/=2 SD below the mean [80]. This signifies that monitoring with cardiac MRI can be beneficial for the patients in the early identification of the long-term cardiotoxic effects of chemotherapy. 

Management and prevention

Management

The management of anthracycline-induced cardiomyopathy is based upon abnormalities in LV preload and afterload and the subsequent progression of fibrosis to the cardiac tissue. This is achieved by medications that target LV preload (diuretics) and LV afterload (angiotensin-converting-enzyme inhibitors or angiotensin receptor blockers) at the initial stages and prevent pathologic LV remodeling. In the treatment of acute heart failure and possible cardiogenic shock, potent drug regimens include diuretics (to reduce high volume states), inotropes (for improvement of contractility of the heart), vasopressors, vasodilators, and drugs involved in calcium sensitization [70]. Investigating the type of cardiomyopathy leading to heart failure is crucial. Anthracyclines cause structural changes in the heart resulting in progression from dilated to a restrictive type of cardiomyopathy. Depending upon the hemodynamic monitoring, it is necessary that symptomatic patients receive precise therapy as a treatment for heart failure from dilated cardiomyopathy varies from the heart failure due to restrictive cardiomyopathy [70].

Dexrazoxane is a potent drug used to lower the cardiotoxicity associated with anthracycline-based chemotherapy for cancer. Dexrazoxane functions by decreasing ROS generation and iron-complex formation from anthracycline therapy [81]. One mechanism that aids in the restoration of LV function post-management with anthracyclines is the growth hormone (GH). GH has been found to lower the stress created on the systolic motion of the LV wall, thus improving the overall performance of LV via the promotion of LV hypertrophy [82]. It has been found that the infusion of anthracycline therapy for six or more hours lowers clinical heart failure risk (in symptomatic patients). Also, there is potential in reduction of the risk of subclinical heart failure (heart failure seen on ECHO in asymptomatic patients) [83].

Patients managed with 5 FU must be observed closely for myocardial ischemia by doing serial EKGs. 5 FU should be immediately withdrawn if the patient has acute chest pain until the diagnostic workup is done. Treatment with coronary vasodilators, such as nitrates and calcium-channel blockers, must be considered [84]. Studies have shown that nitrates and calcium-channel blockers have been used in the management of angina induced by 5 FU [85].

One study has shown that in patients receiving chemotherapy the possibility of cardiotoxicity must be identified before starting the treatment. Screening patients with cardiovascular risk factors or prior history of cardiac toxicity should be performed. Methods of continuous cardiac monitoring include; measuring serum electrolytes and cardiac-related enzymes, baseline, and regular echocardiographic studies, and angiography [68]. In childhood cancer survivors, it is likely for heart failure to quickly become refractory to medical management. In such cases, clinicians should consider the use of alternate mechanical assist devices such as pacemakers and implantable pulsatile or continuous-flow ventricular assist devices. Implantable defibrillators and extracorporeal membrane oxygenation have also proven to help. Heart transplantation can be considered for end-stage anthracycline cardiomyopathy in some patients who are not improving with medical management [70].

Prevention

Cardiotoxicity is a serious and common negative consequence of receiving intense oncological treatments. This adverse effect results in damage to heart tissue, many times leading to death even after surviving the diagnosis of cancer. For this reason, prevention and management methods have been underway and must be investigated further to alleviate those affected. One way to avert this unfortunate impairment of the heart is the utilization of less cardiotoxic drugs [70]. One option is liposomal-encapsulated drugs. A common but toxic drug treatment is doxorubicin, an anthracycline. Encapsulating this drug reduces the concentration the body is exposed to at once, which in turn lessens the probability of cardiotoxicity. Although anthracyclines, in general, are cardiotoxic, some are less than others. For example, epirubicin, idarubicin, and mitoxantrone are less harmful than doxorubicin and can be used as alternatives [4]. This may be attributed to the fact that each drug is metabolized uniquely. For instance, epirubicin is subject to glucuronidation while doxorubicin is not. More importantly, each drug interacts distinctly with cardiac tissue, accounting for the differences seen in the effects of each drug [86]. Derivatives of imatinib, a tyrosine kinase inhibitor, have been adopted to manage chronic myeloid leukemia (CML) because of its efficiency in treatment and diminished degree of toxicity. This advantage is due to imatinib’s characteristic attack on the BCR-ABL gene that results from CML [70].

Drugs may be used to prevent and/or delay cardiac tissue damage from cancer treatments. Dexrazoxane is an iron chelation drug that counters sensitivity to anthracycline-induced cardiotoxicity caused by excess iron [11]. This medication is the only one approved by the U.S. Food and Drug Administration as an effective cardio protectant [4,7]. A study by Martin et al. demonstrated the use of an analog of dexrazoxane, topoisomerase II-inactive 3-carbon linker bisdioxopiperazine, to reduce cardiotoxicity, which supported the belief of the mechanism of dexrazoxane being related to the regulation of cardiomyocyte Top2β [87]. Angiotensin-converting enzyme inhibitors (ACEI) have been used to delay cardiotoxicity. Although ACEI may have limitations, they function to alleviate the left ventricle and prevent complete impairment of the heart. Beta-adrenergic blocking drugs intercept sympathetic innervation to the heart, which in turn minimizes cardiac stress. As a result, beta-blockers such as carvedilol have been used to preserve cardiac health during cancer treatments [4]. Mercaptoethylene sulfonate (Mesna) is a medication taken prophylactically when taking cyclophosphamide, an immunosuppressive drug that results in cardiotoxicity. In a study comparing rats that were administered Mesna before and after treatment with cyclophosphamide and rats that received no medication during treatment with cyclophosphamide, it was observed that most that did receive Mesna lived longer. Further research is needed to understand exactly how Mesna is able to provide such protection, but it is believed that such results could be because this medication is taken up by cardiac cells or it protects stems from the free sulfhydryl [88].

A randomized study conducted in 50 patients with ALL (aged 6 to 12), revealed that treatment with ADR reduced the systolic dysfunction of the left ventricle. When children are treated with ADR, it causes a significant rise in cardiac biomarkers such as plasma troponin I and LDH leading to cardiotoxicity. To mitigate this rise, the study determined that pre-treatment with carvedilol in children taking ADR inhibited the rise in plasma troponin and LDH. Despite the fact that carvedilol’s cardioprotective effect is not fully understood, it may be explained by the drug’s antioxidant properties [9]. When asked how we as physicians can mitigate the cardiotoxic effects of chemotherapeutic drugs, we must analyze each individual preventative strategy and determine if combination therapy can prove to be effective. The study conducted by El-Shitany et al. (2012), showed promising effects of carvedilol on decreasing troponin I and LDH. It is possible that this preventative therapy can be combined with other preventative strategies such as physical exercise in order to minimize the cardiotoxic effects of these essential chemotherapeutic regimens [9]. For children with a deficiency of growth hormone, fulfilling that scarcity has also been observed to prevent cardiotoxicity. Diuretics such as furosemide can alleviate hypervolemia and inotropes such as dopamine can aid in contraction. Furthermore, vasopressors, vasodilators, and calcium sensitizers assist in proper function and circulation throughout the cardiovascular system [70]. Taking the proper medications prophylactically or after treatment may relieve cardiac malfunctions.

Genetics are also involved in the extent to which cancer survivors experience cardiotoxicity. Several gene variants and SNPs have been observed to modulate the amount of damage an individual will experience after treatment. For instance, the SLC28A3 gene, which encodes a transporter in the heart, has the variant rs78537585, which is correlated with reduced susceptibility to anthracycline-induced cardiotoxicity. Nitric oxide synthase (NOS3) is associated with drug metabolism. The NOS3 variant rs1799983 has been shown to protect against cardiotoxicity by way of protecting the heart’s ejection fraction during treatment [11]. Another NOS3 variant, G-894 T, also provides protection by way of increasing the left ventricular ejection fraction [4]. ATP2B1 codes for an ATPase, or a calcium pump, in the phospholipid bilayer. Doxorubicin impedes calcium flow; however, variant rs17249754 is known to protect against cardiotoxicity. As does variant rs932764 in phospholipase C ε (PLCE1) by minimizing the production of ROS. Catalase (CAT) is an enzyme whose function is to convert hydrogen peroxide into water; therefore, protecting cells from hydrogen peroxide’s harmful oxidative state. Possessing the CC allele for variant rs10836235 provides some level of protection from cardiotoxicity. Hyaluronan synthase-3 (HAS3) is a gene that gives rise to molecules that provide protection after trauma to the tissue. The HAS3 variant rs2232228 with the GG genotype has been found to provide more resistance to cardiotoxicity than those with the AA genotype. Similarly, CT and TT genotypes provide more protection against cardiotoxicity than the CC genotype in CUGBP Elav‐like family member 4 (CELF4) rs1786814, a protein involved in pre-mRNA splicing [11]. From what has been discussed, genetic screenings can benefit cancer patients receiving treatment, as knowing the variants and alleles each possess could prepare for what is needed to prevent drug-related cardiotoxicity.

As with many illnesses, an individual’s lifestyle contributes to their overall health. Drugs, alcohol, smoking, obesity, and other underlying diseases, especially cardiovascular ones, may affect the way one tolerates a malfunction in the body. Aiding in the termination of these endangering behaviors and taking note of risk factors in the medical history of a patient may diminish the probability or severity of cardiotoxicity [70]. On the other hand, healthy behaviors such as exercise may be of benefit to a patient in tolerating vigorous treatments. In fact, one study mentions that children who have survived Hodgin lymphoma and engaged in strenuous exercise have been observed to be less vulnerable to cardiovascular diseases than those who were more sedentary. Therefore, promoting healthy habits and exercise to a patient is advantageous [7]. Continuous monitoring of social determinants of health can allow for overall improved clinical outcomes.

## Conclusions

It is evident that oncological pathologies and management in the pediatric population are vastly different than that of the adult population. Reviewing some of the most used chemotherapeutic drugs associated with cardiotoxic side effects is crucial to understanding how we, as clinicians, can better serve this population. A delicate balance must exist between the chemotherapeutic regimens and their cardiotoxic side effects. Effective monitoring with various modalities like cardiac biomarkers, ECG, ECHO, and cardiac MRI can help in the early detection of cardiotoxicity. Aiming to catch the earlier symptoms of negative effects on the pediatric heart can lead to prevention, reversal, or potentially slowing down the deterioration of the overall function and structure of the cardiovascular system. When patients develop symptoms, it is also important to initiate early treatment and reduce progressive worsening of the symptoms. There have been several formulations including liposomal doxorubicin which is considered less cardiotoxic than doxorubicin, which is continuing to be developed as alternatives. Further, advances like pre-treatment with dexrazoxane have been shown to lower the risk of anthracycline-induced CHF.

It is crucial for clinicians to be aware of the cardiotoxic side effects for the treatment of pediatric cancers, the available alternatives, and ways in which we can detect cardiotoxic side effects until we can eliminate them completely. In the meantime, implementing lifestyle modifications can mitigate the risk of progression of cardiac disease. Additional long-term prospective studies are required to completely grasp the risks of cardiac events in the pediatric population and develop strategies to treat them.
